# Systemic and Intravitreal Antagonism of the TNFR1 Signaling Pathway Delays Axotomy-Induced Retinal Ganglion Cell Loss

**DOI:** 10.3389/fnins.2019.01096

**Published:** 2019-10-15

**Authors:** Fernando Lucas-Ruiz, Caridad Galindo-Romero, Manuel Salinas-Navarro, María Josefa González-Riquelme, Manuel Vidal-Sanz, Marta Agudo Barriuso

**Affiliations:** ^1^Grupo de Oftalmología Experimental, Instituto Murciano de Investigación Biosanitaria Virgen de la Arrixaca (IMIB-Arrixaca), Murcia, Spain; ^2^Departamento de Oftalmología, Facultad de Medicina, Universidad de Murcia, Murcia, Spain

**Keywords:** optic nerve crush, retinal ganglion cells, R7050, BDNF, combinatory therapy, neuroprotection

## Abstract

Here, we have blocked the signaling pathway of tumor necrosis factor α (TNFα) in a mouse model of traumatic neuropathy using a small cell permeable molecule (R7050) that inhibits TNFα/TNF receptor 1 (TNFR1) complex internalization. Adult pigmented mice were subjected to intraorbital optic nerve crush (ONC). Animals received daily intraperitoneal injections of R7050, and/or a single intravitreal administration the day of the surgery. Some animals received a combinatorial treatment with R7050 (systemic or local) and a single intravitreal injection of brain derived neurotrophic factor (BDNF). As controls, untreated animals were used. Retinas were analyzed for RGC survival 5 and 14 days after the lesion i.e., during the quick and slow phase of axotomy-induced RGC death. qPCR analyses were done to verify that *Tnfr1* and *TNF*α were up-regulated after ONC. At 5 days post-lesion, R7050 intravitreal or systemic treatment neuroprotected RGCs as much as BDNF alone. At 14 days, RGC rescue by systemic or intravitreal administration of R7050 was similar. At this time point, intravitreal treatment with BDNF was significantly better than intravitreal R7050. Combinatory treatment was not better than BDNF alone, although at both time points, the mean number of surviving RGCs was higher. In conclusion, antagonism of the extrinsic pathway of apoptosis rescues axotomized RGCs as it does the activation of survival pathways by BDNF. However, manipulation of both pathways at the same time, does not improve RGC survival.

## Introduction

The link between tumor necrosis factor-α (TNFα) and retinal ganglion cell (RGC) loss in glaucoma and traumatic optic neuropathies has been extensively investigated in humans (Yuan and Neufeld, 2000; Tezel et al., 2001) and animal models (Agudo et al., 2008; Tezel, 2008; Roh et al., 2012; Cueva Vargas et al., 2015; Tse et al., 2018; Wei et al., 2019).

TNFα is a multi-functional cytokine that promotes inflammation and mediates cell death through the binding to TNF receptor 1 (TNFR1), a death receptor that triggers the extrinsic pathway of apoptosis (Lorz and Mehmet, 2009; Cabal-Hierro and Lazo, 2012; Sedger and McDermott, 2014). In the retina, TNFα is expressed by microglial cells (Yuan and Neufeld, 2000; Roh et al., 2012), and TNFR1 by RGCs (Tezel et al., 2001; Agudo et al., 2008).

TNFα induced cell death does not occur in healthy cells, but in stressed or metabolically imbalanced ones (reviewed in Sedger and McDermott, 2014). Thus, TNFα/TNFR1 signaling depends on the cellular context and can lead to proliferation, apoptosis, or necroptosis. TNFα binding to TNFR1 causes the formation of two multiprotein complexes. Complex I prevents apoptosis, while complex II induces cell death. Complex I is membrane bound, and complex II is activated after being endocytosed and released from the cytoplasmic membrane (reviewed in Cabal-Hierro and Lazo, 2012).

In pre-clinical models of ocular hypertension, the main risk factor of glaucoma, or of traumatic optic nerve injury (optic nerve crush or transection) both, the blockade of TNFα using decoy-receptors (Roh et al., 2012; Tse et al., 2018) or natural anti-TNFα compounds (Kyung et al., 2015), and the deletion of TNFR1 (Tezel et al., 2004) have neuroprotective properties on the injured RGCs.

Anti-TNFα therapies based on decoy-receptors or anti-TNF antibodies have some intrinsic problems such as availability, potential antigenicity, and tissue distribution.

R7050 is a small cell permeable triazoloquinoxaline that blocks TNFα signaling through the inhibition of the ligand-induced TNFR1 endocytosis (Gururaja et al., 2007), impairing the association of TNFR1 with the intracellular adaptor molecules that form the multi-protein complexes. Thus, R7050 is a selective antagonist of the TNFα/TNFR1 pathway. Importantly, R7050 crosses the blood brain barrier and it has been shown that attenuates neurovascular injury and improves neurological behavior in an animal model of intra-cerebral hemorrhage (King et al., 2013).

Here we have tested the neuroprotective potential of R7050 on axotomized RGCs. Because single therapies are not sufficient to stop RGC degeneration (Harvey, 2007) we assayed the TNFR1 antagonist alone or in combination with brain derived neurotrophic factor (BDNF), to date the best administered neuroprotectant for injured RGCs (Mansour-Robaey et al., 1994; Peinado-Ramon et al., 1996; Di Polo et al., 1998; Pernet and Di, 2006; Parrilla-Reverter et al., 2009; Sanchez-Migallon et al., 2011, 2016; Galindo-Romero et al., 2013b; Valiente-Soriano et al., 2015; Feng et al., 2016, 2017; Osborne et al., 2018). BDNF activates survival pathways [PI_3_K/Akt and ERK; (Isenmann et al., 1998; Klocker et al., 2000; Nakazawa et al., 2002)] and TNFR1 antagonism inhibits apoptosis. Thus, the combinatory treatment directed to increase pro-survival pathways and to decrease apoptotic ones would, ideally, have a synergistic effect.

We have used the well-characterized model of optic nerve crush (ONC) in mice (Galindo-Romero et al., 2011, 2013b; Sanchez-Migallon et al., 2016, 2018). The course of RGC death induced by ONC in mice occurs in two phases, a quick one that lasts 7–9 days during which ~85% of RGCs die, and a second slower one thereafter. In this model, 50% of RGCs are lost at day 5 post-lesion (reviewed in Vidal-Sanz et al., 2017). Here we have administered R7050 intravitreally and/or intraperitoneally, and BDNF intravitreally, and analyzed RGC survival at 5 or 14 days post-ONC, i.e., during the quick and the slow phase of death.

## Materials and Methods

### Animal Handling

Adult pigmented C57Bl/6 male mice (25 g) were obtained from the University of Murcia breeding colony. All animals were treated in compliance with the European Union guidelines for Animal Care and Use for Scientific Purpose (Directive 2010/63/EU) and the guidelines from the Association for Research in Vision and Ophthalmology (ARVO) Statement for the Use of Animals in Ophthalmic and Vision Research. All procedures were approved by the Ethical and Animal Studies Committee of the University of Murcia, Spain (number: A1320140704).

Animals undergoing surgery were anesthetized by intraperitoneal injection of a mixture of ketamine (60 mg/kg; ketolar, Pfizer, Alcobendas, Madrid, Spain) and xylazine (10 mg/kg; Rompum, Bayer, Kiel, Germany). Analgesia was provided by subcutaneous administration of buprenorphine (0.1 mg/kg; Buprex, Buprenorphine 0.3 mg/mL; Schering-Plow, Madrid, Spain). During and after surgery, the eyes were covered with an ointment (Tobrex; Alcon S.A., Barcelona, Spain) to prevent corneal desiccation. Animals were sacrificed with an intraperitoneal injection of an overdose of sodium pentobarbital (Dolethal, Vetoquinol; Especialidades Veterinarias, S.A., Alcobendas, Madrid, Spain).

### Animal Groups

**Figure 1:**
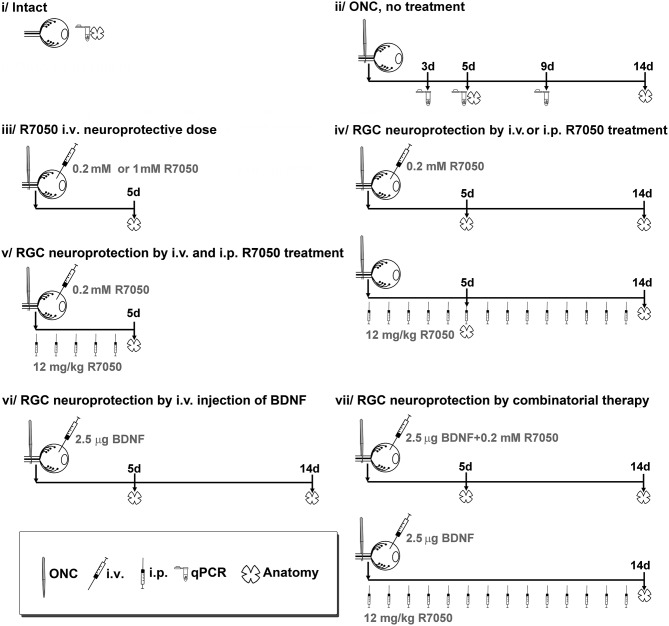
Experimental design and animal groups. R7050 toxicity was studied at 0.2 mM in intact retinas, which were analyzed 5 days after administration. As vehicle control groups, we assayed R7050 1 mM vehicle (5% DMSO in saline) since it has been published that at this concentration is toxic for neurons (Galvao et al., 2014). Two groups were done, intact + i.v. of 5% DMSO and ONC + i.v. of DMSO analyzed 5 days later. To reduce the number of animals, control groups for 1% DMSO (R7050 0.2 mM vehicle) were not done since in our conditions neither the population of RGCs nor their loss after axotomy was affected by an i.v. injection of 5% DMSO. Likewise, intraperitoneal vehicle of R7050 and intravitreal vehicle of BDNF groups were not done because we have already shown that those vehicle formulations and administration routes do not have a negative effect in the retina (Galindo-Romero et al., 2013b; Di Pierdomenico et al., 2018). i.v, intravitreal injection; i.p., intraperitoneal injection.

Animal groups are detailed in Figure 1. The number of retinas per group and time point was 4–6 for anatomical analysis (see Table 1 for a detailed n), and 4 for qPCR.

**Table 1:** Total number of RGCs.

		**Intact**	**ONC**	
			**5d**	**14d**
No treatment	Mean	43,609	22,832	3,782
	SD	1,237	2,362	585
	*n*	6	6	5
i.p. R7050	Mean		25,802^*^	6,993**^†^
	SD		1,002	1,064
	*n*		5	5
i.v. 5% DMSO	Mean	43,935	23,374	
	SD	773	252	
	*n*	4	4	
i.v. 0.2 mM R7050	Mean	43,683	28,911***^##^	5,371^*^^†^
	SD	1,355	1604	942
	*n*	4	5	5
i.v. 1 mM R7050	Mean		28,207***	
	SD		2,313	
	*n*		4	
i.v. 0.2 mM + i.p. R7050	Mean		29,710***^#^	
	SD		1,827	
	*n*		5	
i.v. 2.5 μg BDNF	Mean		27,201**	10,222***^‡^
	SD		2,275	2,187
	*n*		5	4
i.v. 2.5 μg BDNF + i.v. 0.2 mM R7050	Mean		29,351***	11,392***
	SD		1,812	4,023
	*n*		5	5
i.v. 2.5 μg BDNF + i.p. R7050	Mean			12,109***
	SD			1,926
	*n*			5

Intact retinas treated with 5% DMSO or with 0.2 mM of R7050 were analyzed 5 days after the injection. SD, standard deviation; i.v., intravitreal injection; i.p., intraperitoneal injection;

* Statistically different from ONC alone within the same time point (*p < 0.05; **p < 0.01; ***p < 0.001).

# *Statistical difference with i.p. treatment (##p < 0.001; ^#^p < 0.01)*.

† *Significantly different compared to combinatorial treatments (p < 0.05)*.

‡ *Significantly different compared to i.v. R7050 treatment (p < 0.05). At all time post-lesions and irrespectively of the treatment, the loss of RGCs was significant compared to intact retinas (p < 0.01). T-test to compare two treatments. ANOVA Kruskal-Wallis test with Dunn's multiple comparisons post-hoc test, to analyze the effect of a treatment along time*.

### Surgery

The left optic nerve was crushed at 0.5 mm from the optic disc following previously described methods (Galindo-Romero et al., 2011; Sanchez-Migallon et al., 2016). In brief, to access the optic nerve at the back of the eye, an incision was made in the skin overlying the superior orbital rim, the supero-external orbital contents were dissected, and the superior and external rectus muscles were sectioned. Then, the optic nerve was crushed at 0.5 mm from the optic disc for 10 s using watchmaker's forceps. Before and after the procedure, the eye fundus was observed through the operating microscope to assess the integrity of the retinal blood flow.

### Intravitreal and Intraperitoneal Treatments

Intravitreal injections were all done in a final volume of 2.5 μl, following previously published methods (Galindo-Romero et al., 2013b; Sanchez-Migallon et al., 2016). Animals were treated with single or combinatory intravitreal treatments of R7050 (0.2 or 1 mM, in 1 and 5% of DMSO-saline, respectively. Tocris Bioscience; Bio-Techne R&D Systems. Madrid, Spain) and/or BDNF (2.5 μg in 1% bovine serum albumin-PBS, PreproTech, London, UK).

Systemic treatment with R7050 (12 mg/kg i.p.) was done daily for 7 or 14 days. Some animals received a combinatorial therapy of intravitreal and intraperitoneal treatments. All groups are detailed in Figure 1.

### Immunodetection

Animals were perfused transcardially with 0.9% saline solution followed by 4% paraformaldehyde in 0.1 M phosphate buffer. Retinas were prepared as flat mounts, or eyes were cryo-sectioned. In all experimental groups, RGCs were immunodetected in whole-mounts as reported (Galindo-Romero et al., 2011; Sanchez-Migallon et al., 2016) using mouse α-Brn3a primary antibody (1:500; MAB1585, Merck Millipore; Madrid, Spain). Secondary detection was carried out with donkey α-mouse IgG1-Alexa fluor 594 (1:500; Molecular Probes; Thermo Fisher Scientific, Madrid, Spain). In brief, flat mounted retinas were permeabilized in PBS 0.5% Triton by freezing them for 15 min at −70°C, rinsed in new PBS 0.5% Triton, and incubated overnight at 4°C with the primary antibody diluted in blocking buffer (phosphate buffer saline (PBS) with 2% donkey normal serum and 2% Triton). Then, the retinas were washed three times in PBS and incubated for 2 h at room temperature with the secondary antibody. Finally, they were thoroughly washed in PBS, mounted vitreous side up and covered with antifading solution (Vectashield, Vector laboratories, Palex Medical, Barcelona, Spain).

In retinal cross sections from intact animals or animals processed 5 days after ONC, Tnfr1 (1:100, ab19139 Abcam, Cambridge, UK) and Brn3a (MAB1585, 1:500) were double immunodetected. Secondary antibodies were donkey α-mouse IgG1-Alexa fluor 488 or α-rabbit-Alexa fluor 594 (1:500; Molecular Probes). Briefly, sections were incubated overnight a 4°C with the primary antibodies diluted in blocking buffer. Secondary detection was carried out as in flat-mounts.

### Image Acquisition and Analysis

Images were acquired using an epifluorescence microscope (Axioscop 2 Plus; Zeiss Mikroskopie, Jena, Germany) equipped with a computer-driven motorized stage (ProScan H128 Series; Prior Scientific Instruments, Cambridge, UK) controlled by image analysis software (Image-Pro Plus, IPP 5.1 for Windows; Media Cybernetics, Silver Spring, MD).

Retinal photomontages of Brn3a^+^RGCs were reconstructed from 154 (11 × 14) individual images (Galindo-Romero et al., 2011). The whole population of Brn3a^+^RGCs was quantified automatically and their distribution assessed by neighbor maps using previously reported methods (Galindo-Romero et al., 2013a). Briefly for each RGC its center mass position coordinates (x, y) were measured using the IPP macro language and the data were exported to a spreadsheet (Office Excel 2000; Microsoft Corp., Redmond, WA). Next, the Java (Oracle Corporation, Redwood Shores, California, USA) application described previously (Galindo-Romero et al., 2013a) was used to calculate the number of neighbors around each cell by measuring their euclidean distance to the rest of cells. Those cells closer than the fixed radius (0.22 mm) were counted. Finally, spatial study was used to spatially plot every RGC, and the number of neighbors served to color each one with a color scale representing the number of neighbors for each one. All maps were plotted using SigmaPlot (SigmaPlot 9.0 for Windows; Systat Software, Inc., Richmond, CA, USA).

### qPCR

Fresh dissected retinas were immediately frozen on dry ice (*n* = 4 per group and time point). Total RNA was extracted using Trizol reagent (Thermo Fisher Scientific) and the RNA samples were dissolved in 20 μL Milli-Q water. Total RNA concentration was determined using SimpliNanoTM (GE Healthcare Life Sciences, Madrid, Spain). Complementary DNA amplification was performed according to the instructions provided by the manufacturer SuperScript^TM^ IV VILO^TM^ Master Mix, Thermo Fisher Scientific) using 1 μg total RNA.

Mouse pre-designed SYBR green primers (pair 1) for *Tnfrs1, Tnf*α, and *Hrpt* (housekeeping) were purchased from Sigma Aldrich.

SYBR Premix Ex Taq II (Tli RNaseH Plus, TaKara; Thermo Fisher Scientific) based qPCR was carried out by the Genomic Platform at the IMIB-Arrixaca in a final volume of 5 μl with a primer concentration of 450 nM using the QuantStudio 5 (Applied Biosystems; Thermo Fisher Scientific). Technical triplicates were done for each sample. The Ct values were converted to relative quantification using the 2^ΔΔ*Ct*^ method (Livak and Schmittgen, 2001).

### Statistics

Data were analyzed and plotted with GraphPad Prism v.7 (GraphPad San Diego, USA). Anatomical data are presented as mean ± standard deviation (SD), and qPCR data as mean ± standard error of the mean (SEM). Differences were considered significant when *p* < 0.05. Tests are detailed in results.

## Results

### TNFR1 Expression in Intact and Injured Retinas

**Figure 2:**
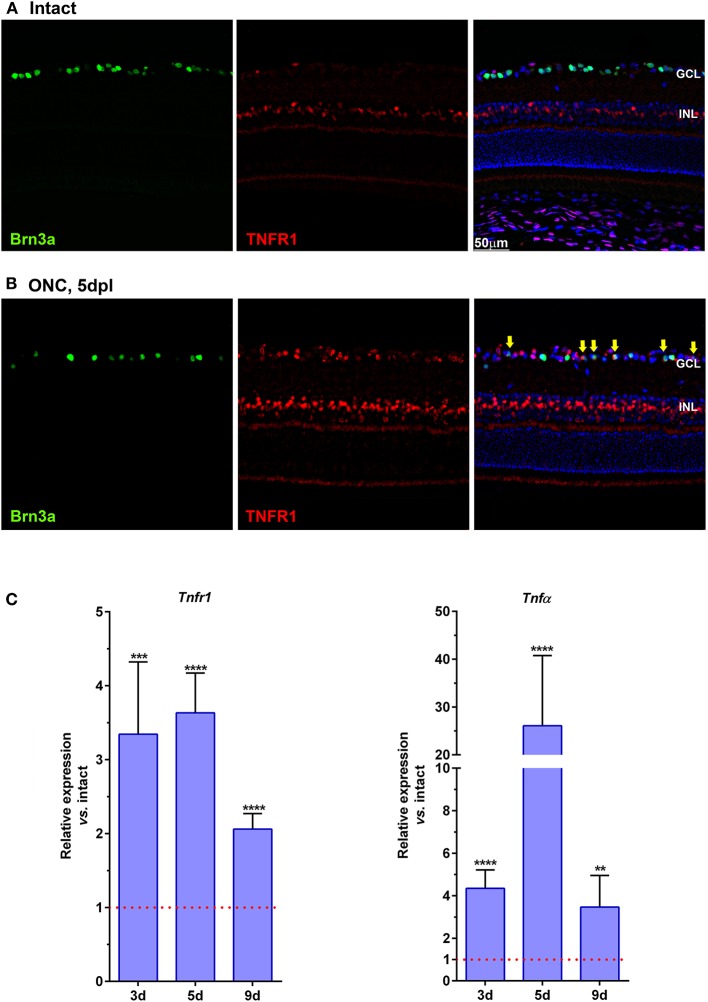
TNFR1 is over-expressed in the retina after ONC. **(A,B)** Magnifications from retinal cross-sections showing the double immunodetection of Brn3a (green), TNFR1 (red), and the merged image with DAPI. In intact retinas **(A)** TNFR1 expression is observed mainly in the inner nuclear layer (INL), and in few Brn3a negative cells in the ganglion cell layer (GCL). Five days after ONC **(B)** many cells in the GCL, including RGCs (yellow arrows) express TNFR1. **(C)** Graph bars showing the fold change ± SEM of *Tnfr1* and *TNF*α mRNA levels in ONC-injured retinas relative to intact retinas (value 1, red dotted line; **p < 0.01; ***p < 0.001; ****p < 0.0001, *T*-test vs. naive). d, days post-lesion.

In intact retinas, TNFR1 is expressed in the inner nuclear layer (INL) and in the ganglion cell layer (GCL) (Figure 2A). TNFR1^+^ cells in the GCL do not express Brn3a, and most possibly are displaced amacrine cells, since they are 50% of the cells in the GCL (Pérez De Sevilla Müller et al., 2007; Nadal-Nicolás et al., 2015). Five days after ONC, TNFR1 is expressed more brightly and by more cells than in intact retinas. In the GCL many RGCs are TNFR1 positive (Figure 2B, yellow arrows). Interestingly, TNFR1 expression is observed in those RGCs with a lower signal of Brn3a, as we observed before for those RGCs expressing the cleaved form of caspase 3 (Sanchez-Migallon et al., 2016).

qPCR analyses support the anatomical data, and at 3, 5, and 9 days after ONC there is a 2–3-fold increase of *Tnfr1* mRNA compared to intact retinas (Figure 2C, left graph). Furthermore, after ONC there is as well a significant increase of the *Tnfr1* ligand, *TNF*α (Figure 2C, right graph), in agreement with previous reports (Tse et al., 2018).

### Intraperitoneal and Intravitreal Antagonism of TNFR1 Rescues RGCs From Optic Nerve Crush

To evaluate if a systemic treatment with R7050 protects axotomized RGCs, we performed ONC and animals were treated daily with an intraperitoneal injection of 12 mg/kg of R7050. This dose was chosen based on a previous work where this antagonist was used to treat the brain (King et al., 2013).

**Figure 3:**
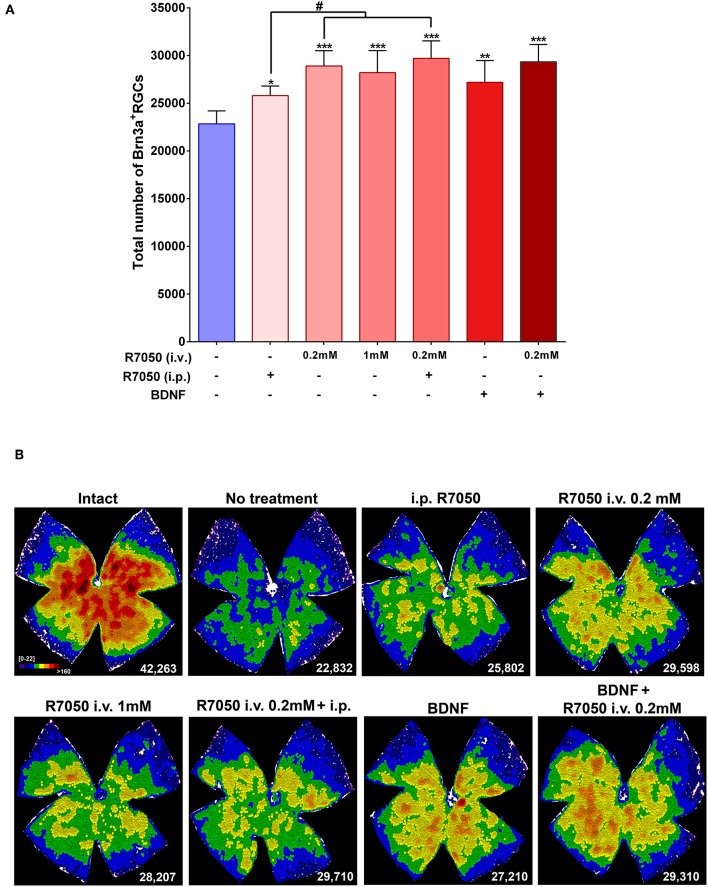
Systemic and intravitreal treatment with R7050 alone or in combination with BDNF rescues RGCs during the quick phase of death after optic nerve axotomy. **(A)** Column bar graph showing the total mean number ± standard deviation of RGCs quantified in the different animal groups analyzed 5 days after ONC. *Statistically different from ONC alone (*p < 0.05; **p < 0.01; ***p < 0.001. ANOVA, Kruskal-Wallis. Dunn's *post-hoc* test). ^#^*p* < 0.01 (*T*-test). For detailed statistics see Table 1. **(B)** Neighbor maps showing the distribution of RGCs in a representative retina from each group and treatment. These maps illustrate the number of RGCs around a given RGC in a radius of 0.200 mm with a color scale (top left panel) from 0 to 22 neighbors (purple) to >160 neighbors (dark red). At the bottom left of each map is shown the number of RGCs counted in the original retina.

**Figure 4:**
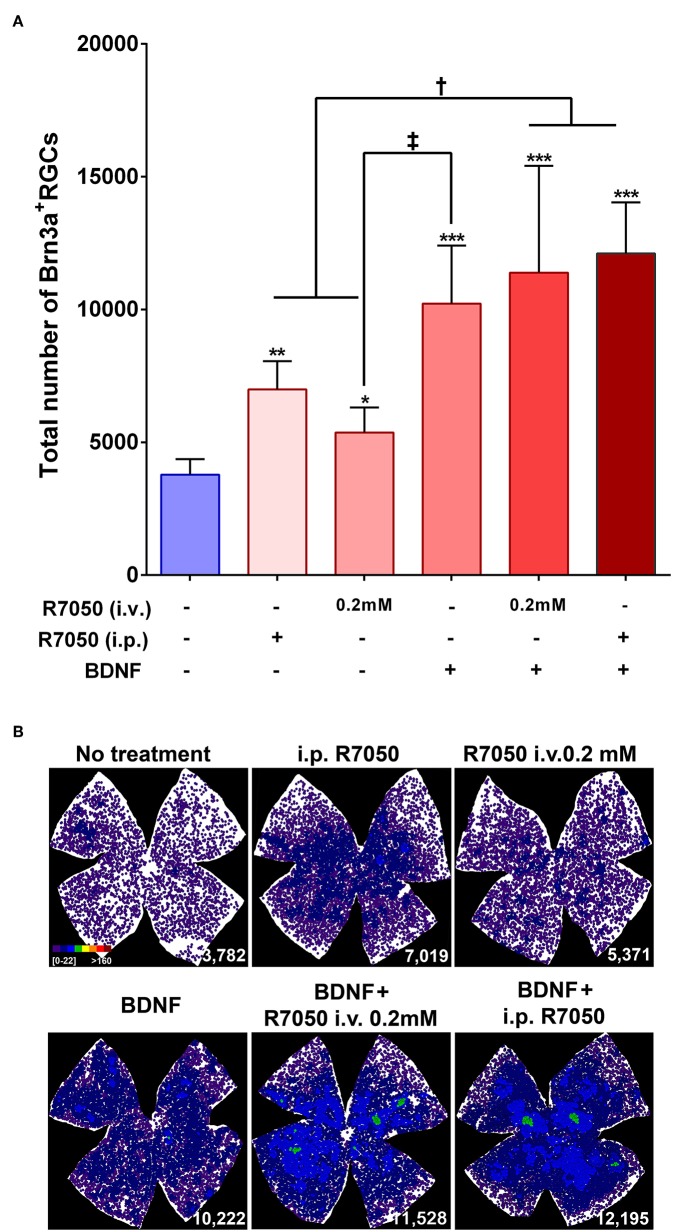
Systemic and intravitreal treatment with R7050 alone or in combination with BDNF rescues RGCs during the slow phase of death after optic nerve axotomy. **(A)** Column bar graph showing the total mean number ± standard deviation of RGCs quantified in the different animal groups analyzed 14 days after ONC. *Statistically different from ONC alone (*p < 0.05; **p < 0.01; ***p < 0.001). ^†^*p* < 0.01; ^‡^*p* < 0.05 (ANOVA, Kruskal-Wallis. Dunn's *post-hoc* test). See Table 1 for more details. **(B)** Neighbor maps showing the distribution of RGCs in a representative retina from each group and treatment. These maps illustrate the number of RGCs around a given RGC in a radius of 0.220 mm with a color scale (top left panel) from 0 to 22 neighbors (purple) to >160 neighbors (dark red). At the bottom left of each map is shown the number of RGCs counted in the original retina.

Five and fourteen days after the lesion, when without treatment 50 and 92%, respectively, of the RGCs have died, the number of RGCs is significantly higher in the treated than in the untreated groups (Table 1; Figures 3, 4).

Next, we wondered whether a single intravitreal treatment would improve this outcome. We tested two different concentrations (0.2 and 1 mM) because this is the first time this route is used for this drug. Since R7050 is dissolved in DMSO, which has been reported toxic for RGCs after intravitreal administration (Galvao et al., 2014), we injected 5% DMSO in PBS (the maximum concentration used here) in intact retinas and after ONC, and both groups were analyzed 5 days later. Our data show that in intact retinas 5% DMSO does not cause RGC loss, and it does not increase the loss of RGCs after ONC (Table 1).

Five days after the lesion, R7050 intravitreal treatment increases significantly the number of surviving RGCs compared to untreated retinas. With the 0.2 mM intravitreal injection, the elicited neuroprotection was significantly higher than after intraperitoneal treatment (Table 1; Figure 3).

Both intravitreal doses achieved a similar RGC rescue and we chose the lower one for subsequent experiments. To verify that the treatment itself was not toxic for RGCs, 0.2 mM R7050 was intravitreally injected in intact retinas. As observed in Table 1, at this dose there was no RGC loss 5 days post-injection. Because the 1 mM dose was not used further and to save animals, we did not test its toxicity.

At 14 days, the intravitreal treatment still protects RGCs (Table 1; Figure 4), but the mean number of surviving RGCs is smaller than with the intraperitoneal treatment, although this difference does not reach statistical significance.

Finally, we combined both routes and treated animals with a single intravitreal injection and a daily intraperitoneal one. This posology does not improve the neuroprotection observed by intravitreal injection alone at 5 days post-lesion, therefore we did not assayed this combination at 14 days (Table 1; Figure 3).

The study of RGC topography by neighbor maps, shows in agreement with previous works, that RGC loss by axotomy affects the whole retina (Galindo-Romero et al., 2011; Nadal-Nicolás et al., 2015; Sanchez-Migallon et al., 2016, 2018). In addition, here we show that the intraperitonal and intravitreal administration of R7050 protects RGCs across the whole retina (Figures 3B, 4B).

In conclusion, at 5 days the percent of surviving RGCs in R7050-treated retinas is 13% (intraperitoneal), 26% (intravitreal, 0.2 mM), 23% (intravitreal, 1 mM), and 30% (intraperitoneal+intravitreal) higher than after ONC alone. Neuroprotection by R7050 is relatively better at 14 than at 5 days: at 14 days there are 54% (intraperitoneal) and 42% (intravitreal) more RGCs than in untreated retinas.

### Combinatorial Treatment: TNFR1 Antagonism and BDNF

BDNF is, excluding knocking out pro-apoptotic proteins or transfecting anti-apoptotic ones (Malik et al., 2005; Nickells et al., 2008), the best neuroprotectant for axotomized RGCs. Thus, we decided to test whether a combinatory therapy with BDNF and R7050 was better than each one alone.

At 5 days after ONC, a single intravitreal injection of BDNF rescues as many RGCs as the intravitreal or intraperitoneal treatment with R7050 (Table 1; Figure 3). An intravitreal injection of BDNF and 0.2 mM of R7050 increases the mean number of surviving RGCs, but no significantly so compared to either treatment alone. To save animals, we did not combine BDNF with systemic R7050, because R7050 administered intravitreally gives better results at this time point.

At 14 days post-injury, RGC neuroprotection by BDNF alone was significantly higher than R7050 administered intravitreally and better, but no significantly, than intraperitoneal R7050. Combination of BDNF and R7050 administered intravitreally or systemically, does not increase significantly the number of surviving RGCs compared to BDNF treatment alone. Even so, and as it occurs at 5 days, the mean number of RGCs is higher in the combinatory experiment (Table 1; Figure 4).

Again, and as observed for the single therapy with R7050, RGC neuroprotection by the combinatory treatments expands the whole retina (Figures 3B, 4B).

In summary, while none of the treatments rescued the whole population of RGCs (see Table 1, intact retinas and neighbor map from an intact retina in Figure 3B), they elicited significant neuroprotection: at 5 days and compared to ONC alone, the percent of surviving RGCs in the treated retinas is 19% (BDNF), and 28% (BDNF + intravitreal R7050) higher. Again neuroprotection is relatively better at 14 than at 5 days: at 14 days there are 270% (BDNF), 301% (BDNF + intravitreal R7050), and 320% (BDNF + intraperitoneal R7050) more RGCs than in untreated retinas.

## Discussion

The role of TNFα in neurodegeneration has been widely reported, not only in the retina (Yuan and Neufeld, 2000; Tezel et al., 2001; Kitaoka et al., 2006; Agudo et al., 2008; Tezel, 2008; Cueva Vargas et al., 2015; De Groef et al., 2015) but also in neurodegenerative diseases such as Alzheimer's or Parkinson's (Mogi et al., 1994; Cheng et al., 2010).

Our results confirm and extend those previous reports connecting axonal damage, RGC loss and TNFα. Our strategy differs from previous works targeting TNFα (Roh et al., 2012; Tse et al., 2018; Park et al., 2019) in that instead of blocking TNFα using decoy-receptors, we have used an antagonist of the TNFα/TNFR1 signaling pathway. R7050 inhibits the endocytosis of the TNF-α/TNFR1 multiprotein complex (Gururaja et al., 2007), thus blocking the extrinsic pathway of apoptosis. This antagonist is a small cell permeable molecule that crosses the blood brain barrier, allowing a systemic administration to treat the central nervous system (King et al., 2013). TNFα decoy receptors are also administered systemically, however because they are proteins they come with inherent problems such as bioavailability, tissue distribution and most importantly, antigenicity.

Our data show that R7050 administered locally or systemically, rescues axotomized RGCs. Intravitreal treatment elicits a better neuroprotection at 5 days than the intraperitoneal route. At long time post-lesions, intraperitoneal and intravitreal administration render similar results. Nevertheless, we should not forget that here we performed a single intravitreal injection, while the systemic dose was administered daily.

Neuroprotection by R7050 14 days after ONC is higher (54–42%) than that shown in a previous work where TNFα was intercepted with etanercept, a decoy-receptor (Tse et al., 2018). Tse et al. reported an increase of ~24% of RGC survival. These differences could mean that R7050 is a better inhibitor of the TNFα apoptotic signaling. Alternatively, the differences could be due to the different route (subcutaneous) or methodology, as they sample the retinas and present RGC densities, while here we quantify the total population of RGCs.

Another strategy to study the implication of a given protein in axotomy-induced RGC death is the use of knockout mice. In a time course study using TNFR1^−/−^ mice, Tezel et al. (2004) analyzed the retinas from 1 to 6 weeks after ONC and observed, in agreement with our data that compared to wild type animals, RGC neuroprotection in the deficient mice was higher at longer times post-lesion.

RGC loss by axotomy triggers a myriad of signals, encompassing multiple pro-death, and pro-survival pathways (Agudo et al., 2008, 2009; Agudo-Barriuso et al., 2013), and this is believed to be one of the reasons why single treatments are not enough (Harvey, 2007). Thus, combinatory therapies are needed to increase neuroprotection not only in number of neurons, but also in time. Here we combined BDNF, whose neuroprotective potential has been widely studied (Mansour-Robaey et al., 1994; Peinado-Ramon et al., 1996; Di Polo et al., 1998; Pernet and Di, 2006; Parrilla-Reverter et al., 2009; Sanchez-Migallon et al., 2011, 2016; Galindo-Romero et al., 2013b; Valiente-Soriano et al., 2015; Feng et al., 2016, 2017; Osborne et al., 2018) with R7050. We hypothesized that activating pro-survival pathways while inhibiting pro-apoptotic ones would increase RGC survival. However, our data show that the combined rescue of R7050 and BDNF is not summative. This suggest that either both pathways share a common denominator, and/or that the activation of the survival pathways by BDNF blocks the apoptotic signals thus effectively overriding the effect of R7050. Further experiments are needed to assess whether a sequential treatment first with BDNF and then with R7050 (or vice versa) would be more effective.

In conclusion, here we show for the first time that local and systemic treatment with the small inhibitor of the TNFR1 signaling, R7050, protects RGCs from axotomy-induced degeneration. While knockout or transgenic animals are very useful to assign specific functions to specific proteins, this strategy cannot (yet) be used in human patients. Given that to date there are no therapies to overcome neuronal death, proof of concept pre-clinical studies are needed to expand the possible therapeutic avenues to treat not only traumatic neuropathies, but also neurodegenerative diseases.

## Data Availability Statement

All datasets generated for this study are included in the manuscript/supplementary files.

## Ethics Statement

The animal study was reviewed and approved by the Ethical and Animal Studies Committee of the University of Murcia, Spain (number: A1320140704).

## Author Contributions

FL-R, CG-R, MS-N, MG-R: methodology, data analysis and representation, and review and editing. MV-S: writing, review and editing, and funding acquisition. MA: conceptualization, supervision, data analysis and representation, writing, review and editing, and funding acquisition.

### Conflict of Interest

The authors declare that the research was conducted in the absence of any commercial or financial relationships that could be construed as a potential conflict of interest.

## References

[B1] Agudo M. Perez-Marin M. C. Lonngren U. Sobrado P. Conesa A. Canovas I.. (2008). Time course profiling of the retinal transcriptome after optic nerve transection and optic nerve crush. Mol. Vis. 14, 1050–1063. 18552980 PMC2426719

[B2] Agudo M. Perez-Marin M. C. Sobrado-Calvo P. Lonngren U. Salinas-Navarro M. Canovas I.. (2009). Immediate upregulation of proteins belonging to different branches of the apoptotic cascade in the retina after optic nerve transection and optic nerve crush. Invest. Ophthalmol. Vis. Sci. 50, 424–431. 10.1167/iovs.08-2404 18775855

[B3] Agudo-Barriuso M. Lahoz A. Nadal-Nicolas F. M. Sobrado-Calvo P. Piquer-Gil M. Diaz-Llopis M.. (2013). Metabolomic changes in the rat retina after optic nerve crush. Invest. Ophthalmol. Vis. Sci. 54, 4249–4259. 10.1167/iovs.12-11451 23696609

[B4] Cabal-Hierro L. Lazo P. S. (2012). Signal transduction by tumor necrosis factor receptors. Cell Signal. 24, 1297–1305. 10.1016/j.cellsig.2012.02.006 22374304

[B5] Cheng X. Yang L. He P. Li R. Shen Y. (2010). Differential activation of tumor necrosis factor receptors distinguishes between brains from Alzheimer's disease and non-demented patients. J. Alzheimers Dis. 19, 621–630. 10.3233/JAD-2010-1253 20110607 PMC3746510

[B6] Cueva Vargas J. L. Osswald I. K. Unsain N. Aurousseau M. R. Barker P. A. Bowie D.. (2015). Soluble tumor necrosis factor alpha promotes retinal ganglion cell death in glaucoma via calcium-permeable ampa receptor activation. J. Neurosci. 35, 12088–12102. 10.1523/JNEUROSCI.1273-15.2015 26338321 PMC6605307

[B7] De Groef L. Salinas-Navarro M. Van Imschoot G. Libert C. Vandenbroucke R. E. Moons L. (2015). Decreased TNF levels and improved retinal ganglion cell survival in MMP-2 null mice suggest a role for MMP-2 as TNF sheddase. Mediators Inflamm. 2015:108617. 10.1155/2015/108617 26451076 PMC4586990

[B8] Di Pierdomenico J. Scholz R. Valiente-Soriano F. J. Sánchez-Migallón M. C. Vidal-Sanz M. Langmann T.. (2018). Neuroprotective effects of FGF2 and minocycline in two animal models of inherited retinal degeneration. Invest. Ophthalmol. Vis. Sci. 59, 4392–4403. 10.1167/iovs.18-24621 30193320

[B9] Di Polo A. Aigner L. J. Dunn R. J. Bray G. M. Aguayo A. J. (1998). Prolonged delivery of brain-derived neurotrophic factor by adenovirus-infected Muller cells temporarily rescues injured retinal ganglion cells. Proc. Natl. Acad. Sci. U.S.A. 95, 3978–3983. 10.1073/pnas.95.7.3978 9520478 PMC19948

[B10] Feng L. Chen H. Yi J. Troy J. B. Zhang H. F. Liu X. (2016). Long-term protection of retinal ganglion cells and visual function by brain-derived neurotrophic factor in mice with ocular hypertension. Invest. Ophthalmol. Vis. Sci. 57, 3793–3802. 10.1167/iovs.16-19825 27421068 PMC4961002

[B11] Feng L. Puyang Z. Chen H. Liang P. Troy J. B. Liu X. (2017). Overexpression of brain-derived neurotrophic factor protects large retinal ganglion cells after optic nerve crush in mice. eNeuro. 4:ENEURO.0331-16.2016. 10.1523/ENEURO.0331-16.2016 28101532 PMC5240030

[B12] Galindo-Romero C. Aviles-Trigueros M. Jimenez-Lopez M. Valiente-Soriano F. J. Salinas-Navarro M. Nadal-Nicolas F.. (2011). Axotomy-induced retinal ganglion cell death in adult mice: quantitative and topographic time course analyses. Exp. Eye Res. 92, 377–387. 10.1016/j.exer.2011.02.008 21354138

[B13] Galindo-Romero C. Jimenez-Lopez M. Garcia-Ayuso D. Salinas-Navarro M. Nadal-Nicolas F. M. Agudo-Barriuso M.. (2013a). Number and spatial distribution of intrinsically photosensitive retinal ganglion cells in the adult albino rat. Exp. Eye Res. 108, 84–93. 10.1016/j.exer.2012.12.010 23295345

[B14] Galindo-Romero C. Valiente-Soriano F. J. Jimenez-Lopez M. Garcia-Ayuso D. Villegas-Perez M. P. Vidal-Sanz M.. (2013b). Effect of brain-derived neurotrophic factor on mouse axotomized retinal ganglion cells and phagocytic microglia. Invest. Ophthalmol. Vis. Sci. 54, 974–985. 10.1167/iovs.12-11207 23307961

[B15] Galvao J. Davis B. Tilley M. Normando E. Duchen M. R. Cordeiro M. F. (2014). Unexpected low-dose toxicity of the universal solvent DMSO. FASEB J. 28, 1317–1330. 10.1096/fj.13-235440 24327606

[B16] Gururaja T. L. Yung S. Ding R. Huang J. Zhou X. McLaughlin J.. (2007). A class of small molecules that inhibit TNFalpha-induced survival and death pathways via prevention of interactions between TNFalphaRI, TRADD, and RIP1. Chem. Biol. 14, 1105–1118. 10.1016/j.chembiol.2007.08.012 17961823

[B17] Harvey A. R. (2007). Combined therapies in the treatment of neurotrauma: polymers, bridges and gene therapy in visual system repair. Neurodegener. Dis. 4, 300–305. 10.1159/000101886 17627133

[B18] Isenmann S. Klocker N. Gravel C. Bahr M. (1998). Short communication: protection of axotomized retinal ganglion cells by adenovirally delivered BDNF *in vivo*. Eur. J. Neurosci. 10, 2751–2756. 10.1046/j.1460-9568.1998.00325.x 9767407

[B19] King M. D. Alleyne C. H. Jr. Dhandapani K. M. (2013). TNF-alpha receptor antagonist, R-7050, improves neurological outcomes following intracerebral hemorrhage in mice. Neurosci. Lett. 542, 92–96. 10.1016/j.neulet.2013.02.051 23499961 PMC3744337

[B20] Kitaoka Y. Kitaoka Y. Kwong J. M. Ross-Cisneros F. N. Wang J. Tsai R. K.. (2006). TNF-alpha-induced optic nerve degeneration and nuclear factor-kappaB p65. Invest. Ophthalmol. Vis. Sci. 47, 1448–1457. 10.1167/iovs.05-0299 16565378

[B21] Klocker N. Kermer P. Weishaupt J. H. Labes M. Ankerhold R. Bahr M. (2000). Brain-derived neurotrophic factor-mediated neuroprotection of adult rat retinal ganglion cells *in vivo* does not exclusively depend on phosphatidyl-inositol-3'-kinase/protein kinase B signaling. J. Neurosci. 20, 6962–6967. 10.1523/JNEUROSCI.20-18-06962.2000 10995840 PMC6772828

[B22] Kyung H. Kwong J. M. Bekerman V. Gu L. Yadegari D. Caprioli J.. (2015). Celastrol supports survival of retinal ganglion cells injured by optic nerve crush. Brain Res. 1609, 21–30. 10.1016/j.brainres.2015.03.032 25813825 PMC4417422

[B23] Livak K. J. Schmittgen T. D. (2001). Analysis of relative gene expression data using real-time quantitative PCR and the 2 (-Delta Delta C (T) method. Methods 25, 402–408. 10.1006/meth.2001.1262 11846609

[B24] Lorz C. Mehmet H. (2009). The role of death receptors in neural injury. Front Biosci. 14, 583–595. 10.2741/3265 19273087

[B25] Malik J. M. Shevtsova Z. Bahr M. Kugler S. (2005). Long-term *in vivo* inhibition of CNS neurodegeneration by Bcl-XL gene transfer. Mol. Ther. 11, 373–381. 10.1016/j.ymthe.2004.11.014 15727933

[B26] Mansour-Robaey S. Clarke D. B. Wang Y. C. Bray G. M. Aguayo A. J. (1994). Effects of ocular injury and administration of brain-derived neurotrophic factor on survival and regrowth of axotomized retinal ganglion cells. Proc. Natl. Acad. Sci. U.S.A. 91, 1632–1636. 10.1073/pnas.91.5.1632 8127857 PMC43217

[B27] Mogi M. Harada M. Riederer P. Narabayashi H. Fujita K. Nagatsu T. (1994). Tumor necrosis factor-alpha (TNF-alpha) increases both in the brain and in the cerebrospinal fluid from parkinsonian patients. Neurosci. Lett. 165, 208–210. 10.1016/0304-3940(94)90746-3 8015728

[B28] Nadal-Nicolás F. M. Sobrado-Calvo P. Jiménez-López M. Vidal-Sanz M. Agudo-Barriuso M. (2015). Long-term effect of optic nerve axotomy on the retinal ganglion cell layer. Invest. Ophthalmol. Vis. Sci. 56, 6095–6112. 10.1167/iovs.15-17195 26393669

[B29] Nakazawa T. Tamai M. Mori N. (2002). Brain-derived neurotrophic factor prevents axotomized retinal ganglion cell death through MAPK and PI3K signaling pathways. Invest. Ophthalmol. Vis. Sci. 43, 3319–3326. 12356841

[B30] Nickells R. W. Semaan S. J. Schlamp C. L. (2008). Involvement of the Bcl2 gene family in the signaling and control of retinal ganglion cell death. Prog. Brain Res. 173, 423–435. 10.1016/S0079-6123(08)01129-1 18929125

[B31] Osborne A. Khatib T. Z. Songra L. Barber A. C. Hall K. Kong G. Y. X.. (2018). Neuroprotection of retinal ganglion cells by a novel gene therapy construct that achieves sustained enhancement of brain-derived neurotrophic factor/tropomyosin-related kinase receptor-B signaling. Cell Death. Dis. 9:1007. 10.1038/s41419-018-1041-8 30258047 PMC6158290

[B32] Park J. Lee S. Y. Shon J. Kim K. Lee H. J. Kim K. A.. (2019). Adalimumab improves cognitive impairment, exerts neuroprotective effects and attenuates neuroinflammation in an Abeta1-40-injected mouse model of Alzheimer's disease. Cytotherapy. 21, 671–682. 10.1016/j.jcyt.2019.04.054 31076196

[B33] Parrilla-Reverter G. Agudo M. Sobrado-Calvo P. Salinas-Navarro M. Villegas-Perez M. P. Vidal-Sanz M. (2009). Effects of different neurotrophic factors on the survival of retinal ganglion cells after a complete intraorbital nerve crush injury: a quantitative *in vivo* study. Exp. Eye Res. 89, 32–41. 10.1016/j.exer.2009.02.015 19268467

[B34] Peinado-Ramon P. Salvador M. Villegas-Perez M. P. Vidal-Sanz M. (1996). Effects of axotomy and intraocular administration of NT-4, NT-3, and brain-derived neurotrophic factor on the survival of adult rat retinal ganglion cells. A quantitative *in vivo* study. Invest. Ophthalmol. Vis. Sci. 37, 489–500. 8595949

[B35] Pérez De Sevilla Müller L. Shelley J. Weiler R. (2007). Displaced amacrine cells of the mouse retina. J Comp Neurol. 505, 177–89. 10.1002/cne.21487 17853452

[B36] Pernet V. Di P. A. (2006). Synergistic action of brain-derived neurotrophic factor and lens injury promotes retinal ganglion cell survival, but leads to optic nerve dystrophy *in vivo*. Brain 129, 1014–1026. 10.1093/brain/awl015 16418178

[B37] Roh M. Zhang Y. Murakami Y. Thanos A. Lee S. C. Vavvas D. G.. (2012). Etanercept, a widely used inhibitor of tumor necrosis factor-alpha (TNF-alpha), prevents retinal ganglion cell loss in a rat model of glaucoma. PLoS. ONE. 7:e40065. 10.1371/journal.pone.0040065 22802951 PMC3388998

[B38] Sanchez-Migallon M. C. Nadal-Nicolas F. M. Jimenez-Lopez M. Sobrado-Calvo P. Vidal-Sanz M. Agudo-Barriuso M. (2011). Brain derived neurotrophic factor maintains Brn3a expression in axotomized rat retinal ganglion cells. Exp. Eye Res. 92, 260–267. 10.1016/j.exer.2011.02.001 21315070

[B39] Sanchez-Migallon M. C. Valiente-Soriano F. J. Nadal-Nicolas F. M. Vidal-Sanz M. Agudo-Barriuso M. (2016). Apoptotic retinal ganglion cell death after optic nerve transection or crush in mice: delayed RGC loss with BDNF or a caspase 3 inhibitor. Invest. Ophthalmol. Vis. Sci. 57, 81–93. 10.1167/iovs.15-17841 26780312

[B40] Sanchez-Migallon M. C. Valiente-Soriano F. J. Salinas-Navarro M. Nadal-Nicolas F. M. Jimenez-Lopez M. Vidal-Sanz M. (2018). Nerve fibre layer degeneration and retinal ganglion cell loss long term after optic nerve crush or transection in adult mice. Exp. Eye Res. 170, 40–50. 10.1016/j.exer.2018.02.010 29452106

[B41] Sedger L. M. McDermott M. F. (2014). TNF and TNF-receptors: from mediators of cell death and inflammation to therapeutic giants - past, present and future. Cytokine Growth Factor Rev. 25, 453–472. 10.1016/j.cytogfr.2014.07.016 25169849

[B42] Tezel G. (2008). TNF-alpha signaling in glaucomatous neurodegeneration. Prog. Brain Res. 173, 409–421. 10.1016/S0079-6123(08)01128-X 18929124 PMC3150483

[B43] Tezel G. Li L. Y. Patil R. V. Wax M. B. (2001). TNF-alpha and TNF-alpha receptor-1 in the retina of normal and glaucomatous eyes. Invest. Ophthalmol. Vis. Sci. 42, 1787–1794. 11431443

[B44] Tezel G. Yang X. Yang J. Wax M. B. (2004). Role of tumor necrosis factor receptor-1 in the death of retinal ganglion cells following optic nerve crush injury in mice. Brain Res. 996, 202–212. 10.1016/j.brainres.2003.10.029 14697498

[B45] Tse B. C. Dvoriantchikova G. Tao W. Gallo R. A. Lee J. Y. Pappas S.. (2018). Tumor necrosis factor inhibition in the acute management of traumatic optic neuropathy. Invest. Ophthalmol. Vis. Sci. 59, 2905–2912. 10.1167/iovs.18-24431 30025145 PMC5989875

[B46] Valiente-Soriano F. J. Nadal-Nicolas F. M. Salinas-Navarro M. Jimenez-Lopez M. Bernal-Garro J. M. Villegas-Perez M. P. (2015). BDNF rescues RGCs but not intrinsically photosensitive RGCs in ocular hypertensive albino rat retinas. Invest. Ophthalmol. Vis. Sci. 56, 1924–1936. 10.1167/iovs.15-16454 25722208

[B47] Vidal-Sanz M. Galindo-Romero C. Valiente-Soriano F. J. Nadal-Nicolas F. M. Ortin-Martinez A. Rovere G.. (2017). Shared and differential retinal responses against optic nerve injury and ocular hypertension. Front. Neurosci. 11:235. 10.3389/fnins.2017.00235 28491019 PMC5405145

[B48] Wei X. Cho K. S. Thee E. F. Jager M. J. Chen D. F. (2019). Neuroinflammation and microglia in glaucoma: time for a paradigm shift. J. Neurosci. Res. 97, 70–76. 10.1002/jnr.24256 29775216 PMC6239948

[B49] Yuan L. Neufeld A. H. (2000). Tumor necrosis factor-alpha: a potentially neurodestructive cytokine produced by glia in the human glaucomatous optic nerve head. Glia 32, 42–50. 10.1002/1098-1136(200010)32:13.3.CO;2-V 10975909

